# A CREB1-miR-181a-5p loop regulates the pathophysiologic features of bone marrow stromal cells in fibrous dysplasia of bone

**DOI:** 10.1186/s10020-021-00341-z

**Published:** 2021-07-22

**Authors:** Yu Fu, Zhili Xin, Ziji Ling, Hanyu Xie, Tao Xiao, Xin Shen, Jialin Lin, Ling Xu, Hongbing Jiang

**Affiliations:** 1 Jiangsu Province Key Laboratory of Oral Diseases, Nanjing Medical University, No.136, Hanzhong Road, Nanjing, 210029 Jiangsu Province China; 2 Department of Oral and Maxillofacial Surgery, Affiliated Hospital of Stomatology, Nanjing Medical University, Nanjing, 210029 China; 3 Jiangsu Province Engineering Research Center of Stomatological Translational Medicine, Nanjing Medical University, Nanjing, 210029 China; 4 Department of Oral and Maxillofacial Surgery, Nanjing Stomatological Hospital, Medical School of Nanjing University, Nanjing, 210008 China

**Keywords:** CREB, miR-181a-5p, Bone marrow stromal cells, Fibrous dysplasia, Proliferation, Apoptosis

## Abstract

**Background:**

Fibrous dysplasia (FD) is a bone marrow stromal cell (BMSC) disease caused by activating mutations of guanine nucleotide-binding protein alpha-stimulating activity polypeptide (GNAS) and is characterized by increased proliferative activity and disrupted osteogenesis of BMSCs. However, the molecular mechanisms regulating the pathophysiologic features of BMSCs in FD remain unknown. This study aimed to identify and verify the roles of the CREB1-miR-181a-5p regulatory loop in FD pathophysiology.

**Methods:**

MicroRNA (miRNA) sequencing analysis was used to identify the possible miRNAs implicated in FD. The proliferation, apoptosis, and osteogenic differentiation of BMSCs, as well as the osteoclast-induced phenotype, were measured and compared after exogenous miR-181a-5p transfection into FD BMSCs or miR-181a-5p inhibitor transfection into normal BMSCs. Chromatin immunoprecipitation and luciferase reporter assays were performed to verify the interactions between CREB1 and miR-181a-5p and their effects on the FD pathological phenotype.

**Results:**

Compared to normal BMSCs, FD BMSCs showed decreased miR-181a-5p levels and exhibited increased proliferative activity, decreased apoptotic capacity, and impaired osteogenesis. FD BMSCs also showed a stronger osteoclast activation effect. miR-181a-5p overexpression reversed the pathophysiologic features of FD BMSCs, whereas miR-181a-5p suppression induced an FD-like phenotype in normal BMSCs. Mechanistically, miR-181a-5p was the downstream target of CREB1, and CREB1 was posttranscriptionally regulated by miR-181a-5p.

**Conclusions:**

Our study identifies that the interaction loop between CREB1 and miR-181a-5p plays a crucial role in regulating the pathophysiologic features of FD BMSCs. MiR-181a-5p may be a potential therapeutic target for the treatment of FD.

**Supplementary Information:**

The online version contains supplementary material available at 10.1186/s10020-021-00341-z.

## Background

Fibrous dysplasia (FD) is a nonhereditary bone disease caused by GNAS gene mutation in bone marrow stromal cells (BMSCs); in FD, normal bone tissue is replaced by overproliferated fibrous tissue and immature trabecular bone (Boyce and Collins 2020). FD progresses slowly, and FD in the craniomaxillofacial region can result in several clinical features, such as local bone swelling, deformity, and dental occlusion dysfunction (Akintoye et al. 2013). Because of the unclear pathogenesis of FD, osteoclastic suppressor-related drugs and surgical intervention are still the main forms of treatment (Feller et al. 2009).

Overaccumulation of cyclic adenosine monophosphate (cAMP) in FD due to guanine nucleotide-binding protein alpha-stimulating activity polypeptide (GNAS) mutation is the key cause of FD pathogenesis (Zhang et al. 2012a; Riminucci et al. 2010). Excessive cAMP resulting from Gαs signaling activates cAMP-response element-binding protein (CREB) via cAMP-dependent protein kinase (PKA) (Mayr and Montminy 2001; Sands and Palmer 2008). Some studies have shown that aberrant gene expression is involved in the abnormal phenotype of FD BMSCs through binding of cAMP with the cAMP-response element (CRE) in the promoter region of these genes and subsequent activation or inhibition (Boyce and Collins 2020; Fan et al. 2012; Khan et al. 2018).

MicroRNAs (miRNAs) are a class of small noncoding RNAs derived from precursors with a hairpin-like structure, and significant insight has been gained regarding their potential importance in numerous biological and pathological processes, such as cell proliferation, apoptosis and differentiation (Huang et al. 2011; Miska 2005; Frohlich 2019; Long et al. 2020). MicroRNAs are encoded in the genome and transcribed by RNA polymerase II (Pol II) (Ha and Kim 2014). Additionally, transcription factors could also bind to miRNA promoter regions to either activate or repress their transcription (Zhang et al. 2015). Most miRNAs regulate target gene expression by binding its 3′-untranslated region (3′UTR) in a sequence-complimentary manner to repress messenger RNA (mRNA) translation or facilitate mRNA degradation (Waki et al. 2016; Luan et al. 2017). Increasing evidence indicates that miRNAs participate in multiple biological processes in many diseases, and modulation of miRNAs has been used for the therapy of different disorders.

The miR-181 family, comprising miR-181a, miR-181b, miR-181c, and miR-181d, which are highly conserved sequences (Braicu et al. 2019). A growing number of studies show that miR-181a is involved in cell differentiation (Pop-Bica et al. 2018; Bhushan et al. 2013), autophagy (Rippo et al. 2014), apoptosis (Ouyang et al. 2012) and proliferation (Braicu et al. 2019). Moreover, miR-181a is reported to regulate the production of intracellular cAMP by decreasing AC9 expression (Zhuang et al. 2014) and to downregulate CREB1 expression by targeting its mRNA 3′ UTR in neurons (Liu et al. 2013).

In the present study, we aimed to explore the miR-181a-5p expression level in FD BMSCs and reveal that low expression of miR-181a-5p leads to increased proliferative ability, decreased apoptotic capacity, and impaired osteogenesis of FD BMSCs and increases the osteoclast differentiation potential. Furthermore, our study demonstrates that the CREB1-miR-181a-5p loop is critically involved in the pathologic mechanism underlying craniofacial FD. These results suggest that therapeutic intervention targeting the CREB1-miR-181a-5p loop might be beneficial for offering novel insight into the clinical treatment of FD.

## Methods

### Primary cell culture

All experiments were performed under a protocol approved by the Ethics and Research Committee of Nanjing Medical University; fresh FD tissues were immediately obtained from bone lesions after surgical removal. As a control, normal jaws were harvested from the maxilla alveoli as previously described (Xiao et al. 2019). Informed consent was obtained before volunteers were enrolled in this study. Primary BMSCs were cultured in 25 cm^2^ flasks with standard medium consisting of Dulbecco’s modified Eagle’s medium (DMEM) (Gibco, Grand Island, NY, USA), 100 U/ml penicillin and 100 μg/ml streptomycin, and 10% fetal bovine serum (FBS) (ScienCell, Carlsbad, CA, USA) at 37 °C maintained in 5% CO_2_. The medium was changed every 3 days until 80–90% confluence was achieved. BMSCs from FD lesions and normal jaws were used at passages 3–5 throughout the experiments.

### miRNA sequencing analysis

Total RNA from BMSCs and FD BMSCs was extracted using TRIzol reagent (Takara, Dalian, China) and used for miRNA sequencing analysis (Aksomics, China).

### KEGG analysis of target genes

Potential target genes of miR-181a-5p were predicted using the TargetScan, miRWalk, miRPathDB and MiRDB databases, with the Micro-T threshold set at 0.8 and the P-value threshold set at 0.05. The common target genes subsequently underwent Kyoto Encyclopedia of Genes and Genomes (KEGG) database analysis to identify the enriched pathways that might be involved.

### miRNA mimics/inhibitor transfection and RNA interference

Cells were cultured in 12-well plates for transfection at a concentration of 8 × 10^4^ cells per well. MiR-181a-5p mimics, inhibitor and control (miR NC) were purchased from GenePharma (Shanghai, China) and transfected into cells using Lipofectamine 2000 (Invitrogen, Carlsbad, CA, USA). A final concentration of 20 nmol/l miRNA was used for miRNA transfection. Small interfering RNA (siRNA) targeting CREB1 and scramble siRNA were purchased from GenePharma (Shanghai, China). The CREB1 siRNA sequence, 5′-GCCACAGAUUGCCACAUUATT-3′, 5′-UAAUGUGGCA AUCUGUGGCTT-3′, followed our previous study (Xiao et al. 2019), and the scramble siRNA sequence was 5′-UUCUCCGAACGUGUCACGUTT-3′, 5′-ACGUGACACGUUCGGAGAATT-3′.

### cAMP extraction and measurement

For measurement of intracellular levels of cAMP, the cells were incubated for 1 h in serum-free medium containing 1 mM 3-isobutyl-1-methylxanthine (Sigma, St. Louis, MO, USA). cAMP was extracted by 0.1 M HCl for 20 min, and the lysate was centrifuged at 1000×*g* for 10 min at 4 °C. The supernatant was collected for measurement of cAMP levels using a Cyclic AMP ELISA Kit (Cayman, Ann Arbor, MI, USA) according to the manufacturer’s instructions. The results were expressed as picomoles/milligram protein (pmol/mg protein).

### Osteoblast differentiation and alizarin red staining

Cells were cultured in complete medium supplemented with 50 μM ascorbic acid (Sigma, St. Louis, MO, USA), 10 mM β-glycerophosphate (Sigma), and 10^–7^ M dexamethasone (Sigma). The medium was changed every 3 days. The mineralization potential was assessed via alizarin red staining when cells were cultured in osteogenic medium for 14 days. For alizarin red staining, the cells were fixed in anhydrous alcohol for 30 min and washed with double-distilled H_2_O. Subsequently, the cells were stained with 2% Alizarin Red S (pH 4.2) (Sigma) for 10 min. To quantify nodule mineralization, calcified nodules were eluted with 10% cetylpyridinium chloride (CPC) (Sigma), and the absorbance at 562 nm was compared to calcium standards.

### Osteoclast differentiation

Conditioned medium was collected from cell cultures. The medium was collected after the cells reached confluence for 24 h, briefly centrifuged and then stored at -80 °C. Bone marrow mononuclear cells (BMMCs) were obtained as described previously (Zhu et al. 2019; Wijekoon et al. 2018). BMMCs were seeded in 48-well plates (Corning, New York, USA) at a density of 10^5^ cells/well and cultured in α-MEM (Gibco) containing 20 ng/ml recombinant human M-CSF (R&D Systems, USA) and 10% FBS for 3 days. After 3 days, nonadherent cells were removed by washing, and adherent cells were further cultured in 80% α-MEM supplemented with 20 ng/ml M-CSF, 50 ng/ml recombinant human RANKL (R&D Systems), 10% FBS and 20% conditioned medium obtained from cell culture supernatants for 10 days. The culture media were changed every three days (Hong et al. 2014).

### Western blot

Western blot was performed as previously described (Xu et al. 2016). Briefly, cells were lysed, and the lysate was separated on 10% SDS-PAGE gels and subsequently transferred to PVDF membranes (Millipore, Billerica, MA, USA). The membranes were blocked in 5% fat-free milk for 2 h and subsequently incubated with different primary antibodies (1:1000 dilution) at 4 °C overnight. Detailed information regarding the primary antibodies is listed in Additional file 1: Table S1. After washing, the membranes were incubated with secondary antibodies (1:5000 dilution). The proteins were detected by ImageQuant LAS 4000 (GE, USA). The expression levels were normalized to those of GAPDH. Quantitative analysis of the western blot was carried out using ImageJ software.

### RNA extraction and quantitative real-time PCR

Total RNA from cells was extracted using TRIzol reagent (Takara) according to the manufacturer's instructions. Quantitative real-time PCR analyses were performed in triplicate using SYBR Green PCR Master Mix (Vazyme, Nanjing, Jiangsu, China), and reactions were detected using an Applied Biosystems 7900HT Fast Real-time PCR system (Applied Biosystems, Gaithersburg, CA, USA). The primer sequences used for quantitative real-time PCR are listed in Additional file 2: Table S2. The expression levels of mRNA were normalized to GAPDH. The primer of miR-181a-5p, miR-145-3p, miR-98-3p, miR-92b-5p and U6 were purchase from GeneCopoeia (Guangzhou, China) and All-in-One™ miRNA RT-qPCR Detection kit (GeneCopoeia) was used to analyze miRNAs expression, with U6 small nucleolar RNA as an internal control.

### Tartrate-resistant acid phosphate (TRAP) staining

Cells were subjected to TRAP staining using a kit (Sigma) following the manufacturer’s instructions. Cells containing more than 3 TRAP-positive nuclei were considered multinuclear osteoclasts and were counted by three independent assessors.

### F-actin ring formation assay

Multinuclear osteoclasts were fixed with 4% formaldehyde for 30 min and permeabilized with 0.2% Triton X-100 for 5 min. The cells were then blocked with 1% goat serum and 3% BSA and incubated with 2 U/ml rhodamine phalloidin (Beyotime, Shanghai, China) (1:1000 dilution) at room temperature for 30 min. Cell nuclei were stained with 1 μg/ml DAPI (Beyotime) for 1 min. Cells were visualized using a fluorescence microscope.

### Bromodeoxyuridine assay and immunofluorescence

Cells were seeded in 24-well at a density of 10^4^ cells per well and cultured until density reached 50%. Then cells were incubated for 24 h with 0.03 mg/ml bromodeoxyuridine (BrdU) (Sigma) and fixed with 4% paraformaldehyde for 30 min. Fixed cells were incubated for 30 min in 2 M HCl, 15 min in 0.1 M boric acid, 15 min in 0.5% Triton X-100, blocked for 1 h with normal goat serum at 37 °C, and incubated with an anti-BrdU antibody (1:300 dilution, Proteintech, USA) at 4 °C overnight according to the manufacturer’s instructions. The cells were then incubated for 40 min with a secondary antibody labeled with Cy3 (1:50 dilution, ABclonal, China) and stained with DAPI. The percentage of BrdU-positive cells in six randomly selected fields was determined using a microscope. The immunofluorescence staining assay was performed as we described previously (Liu et al. 2019).

### Chromatin immunoprecipitation (ChIP) analysis

ChIP analysis was carried out using EZ-ChIP (Millipore) according to the manufacturer’s protocol. Briefly, FD BMSCs were cross-linked with fresh formaldehyde at a final concentration of 1% at room temperature. Glycine was used to terminate the process. Then, the cells were lysed in SDS buffer and sonicated to shear the DNA at 4 °C. Lysates diluted with ChIP dilution buffer were immunoprecipitated with an anti-CREB1 antibody, and a negative rabbit IgG antibody (Proteintech) was used as an internal control. Reverse-crosslinked DNA was transferred and purified and quantified by quantitative real-time PCR analysis. The primer sequences used for quantitative real-time PCR are listed in Additional file 3: Table S3.

### Dual luciferase reporter assay

The putative binding region of miR-181a-5p in the CREB1 3’UTR was amplified by PCR from genomic DNA and cloned downstream of the firefly luciferase gene (FL) in the *pGL3*-basic luciferase reporter vector (Genecopoeia, Guangzhou, Guangdong, China). For the luciferase reporter assay, 293T cells were cotransfected with individual *pGL3-miR* reporter vectors (wild type or site mutated plasmid) and miR-181a-5p mimics or scramble control (miR NC) using Lipofectamine 2000 for 48 h. Cell lysates were collected and assayed with a Dual Luciferase Assay kit (Promega, Madison, WI, USA) following the manufacturer’s instructions. The pRL Renilla luciferase (RL) reporter was used as an internal control. The results are displayed as the ratio of FL/RL activity.

### Statistical analysis

All data examined are presented as the mean ± S.E.M. values. All experiments were repeated independently at least three times. The statistical significance of differences between groups was calculated using Student’s t-test. *P* < 0.05 was considered significant.

## Results

### Identification of downregulated miR-181a-5p in FD BMSCs

**Fig. 1:**
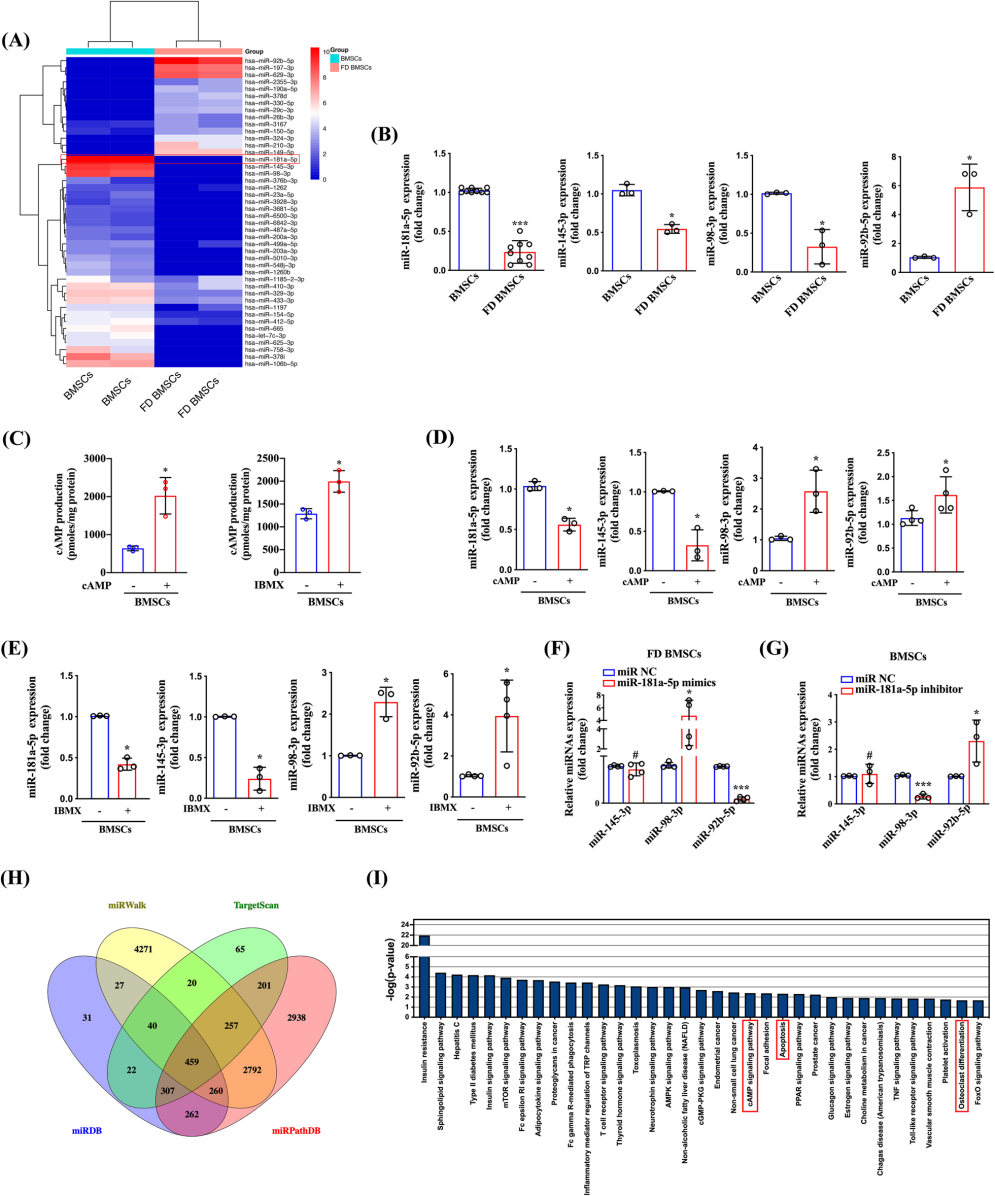
MiR-181a-5p is expressed at low levels in FD BMSCs. **a** MicroRNA sequencing analysis showed the differentially expressed miRNAs between FD BMSCs and BMSCs. **b** Quantitative real-time PCR analysis was used to detect miR-181a-5p, miR-145-3p, miR-98-3p and miR-92b-5p expression between FD BMSCs and BMSCs. **c** cAMP levels in BMSCs treated with cAMP or IBMX were analyzed by ELISA. MiR-181a-5p, miR-145-3p, miR-98-3p and miR-92b-5p expression in BMSCs treated with cAMP (**d**) or IBMX (**e**) was analyzed by quantitative real-time PCR. **f** Quantitative real-time PCR analysis was used to detect miR-145-3p, miR-98-3p and miR-92b-5p expression in FD BMSCs transfected with miR-181a-5p mimics. **g** Quantitative real-time PCR analysis was used to detect miR-145-3p, miR-98-3p and miR-92b-5p expression in BMSCs transfected with miR-181a-5p inhibitor. **h** Venn diagram showing the number of miR-181a-5p target genes predicted by performing miRDB, miRPathDB, miRWalk, and TargetScan algorithms. **i** KEGG analysis of the target genes of miR-181a-5p. The data are presented as the mean ± S.E.M. values (n ≥ 3). ^#^*P* > 0.05; *P < 0.05; ***P < 0.001

Aiming to screen the possible miRNAs implicated in FD, we measured the expression of miRNAs by miRNA sequencing and found that miR-181a-5p was expressed at significantly lower levels in FD BMSCs than in normal BMSCs (fold > 10, *P* < 0.05) (Fig. 1a), whereas miR-145-3p and miR-98-3p were downregulated 9.1 folds and 9 folds in FD BMSCs, respectively. Quantitative real-time PCR further verified this result (Fig. 1b). Based on our previous study, we treated normal BMSCs with 2 mM dibutyryl cAMP (cAMP) (Selleck, Shanghai, China) or 1 mM 3-isobutyl-1-methylxanthine (IBMX) (Sigma), a broad-spectrum phosphodiesterase inhibitor, to increase intracellular cAMP expression to imitate the pathological process of FD BMSCs, and downregulated expression of miR-181a-5p was detected (Fig. 1c–e). Moreover, miR-145a-3p expression was also downregulated with cAMP or IBMX treatment, while miR-98-3p and miR-92b-5p were increased (Fig. 1d, e). To study the effect of miR-181a-5p on miR-145-3p, miR-98-3p and miR-92b-5p, FD BMSCs and BMSCs were transfected with miR-181a-5p mimics and inhibitor, respectively. Quantitative real-time PCR results showed that miR-145-3p had no change with miR-181a-5p mimics or inhibitor treatment, whereas miR-98-3p expression showed a positive correlation with miR-181a-5p abundance, and miR-92b-5p indicated an opposite result (Fig. 1f, g). To explore the possible function of miR-181a-5p in FD, we predicted potential target genes of miR-181a-5p by TargetScan, miRWalk, miRPathDB and MiRDB and found that 459 genes were shared in the above four databases (Fig. 1h). Furthermore, we performed KEGG analysis of the 459 target genes, and the results showed that miR-181a-5p might be involved in several pathways, including the cAMP signaling pathway, apoptosis and osteoclast differentiation (Fig. 1i). These results indicate that miR-181a-5p is expressed at low levels in FD BMSCs and may participate in the pathophysiologic process of FD.

### Low miR-181a-5p expression suppresses the apoptosis and promotes the proliferation of FD BMSCs

**Fig. 2:**
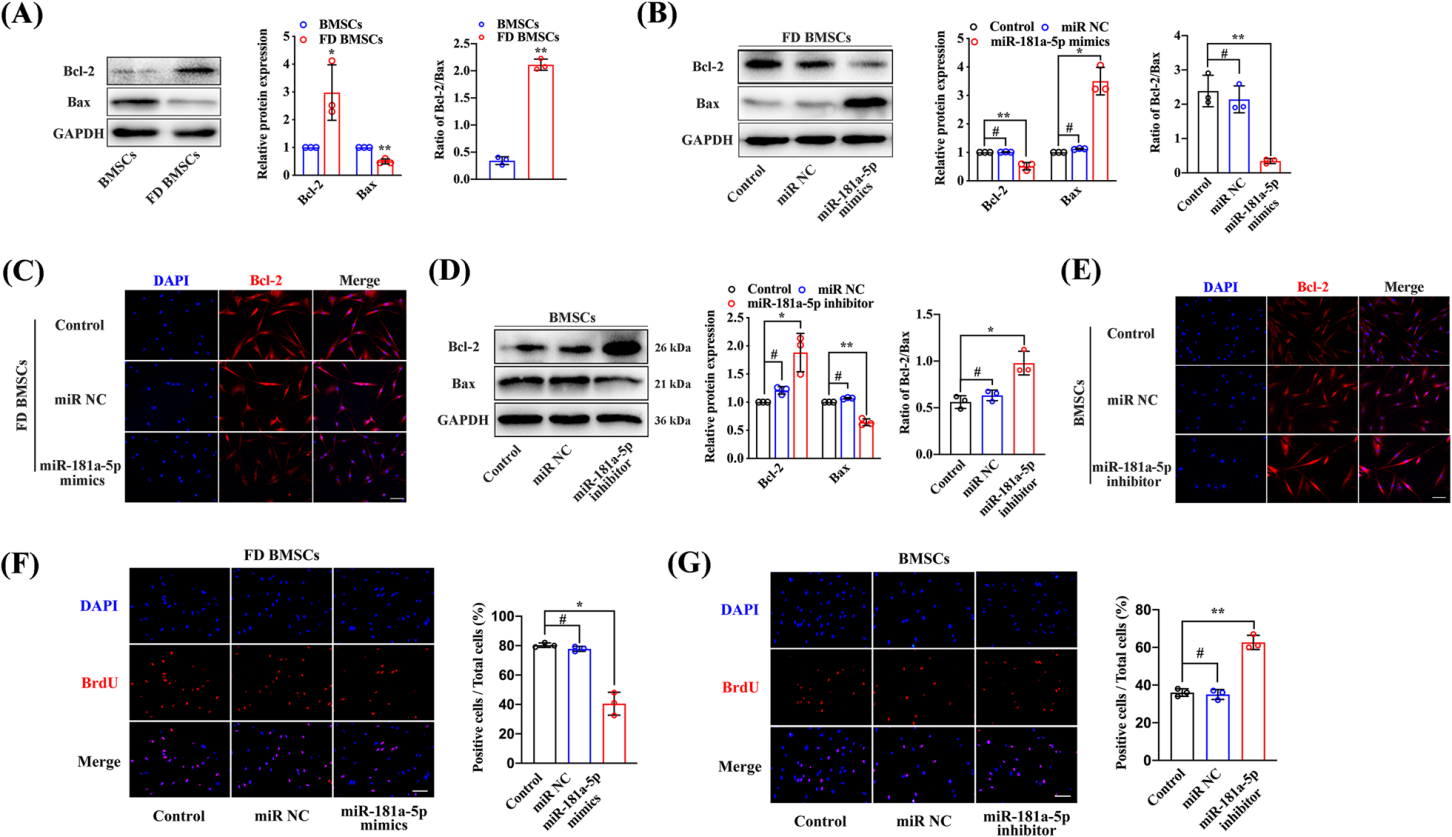
Low miR-181a-5p expression suppresses the apoptosis and induces the proliferation of FD BMSCs. **a** Protein expression of Bcl-2 and Bax in BMSCs and FD BMSCs was detected by western blot. **b** Western blot analysis of Bcl-2 and Bax in FD BMSCs transfected with miR-181a-5p mimics. **c** Immunofluorescence analysis of Bcl-2 expression in FD BMSCs transfected with miR-181a-5p mimics. **d** Western blot analysis of Bcl-2 and Bax in BMSCs transfected with the miR-181a-5p inhibitor. **e** Immunofluorescence analysis of Bcl-2 expression in BMSCs transfected with the miR-181a-5p inhibitor. Representative BrdU incorporation in FD BMSCs transfected with miR-181a-5p mimics (**f**) and BMSCs transfected with the miR-181a-5p inhibitor (**g**). Scale bar, 100 μm. The data are presented as the mean ± S.E.M. values (n = 3). ^#^*P* > 0.05; *P < 0.05; **P < 0.01

Based on our previous study, FD BMSCs exhibited stronger proliferation ability and weaker apoptotic capacity than BMSCs (Xiao et al. 2019). We compared the expression of Bcl-2, a target gene of miR-181a, between FD BMSCs and BMSCs, and upregulated expression of Bcl-2 and downregulated expression of Bax were detected (Fig. 2a). Moreover, the Bcl-2/Bax ratio was increased in FD BMSCs (Fig. 2a). To verify the role of miR-181a-5p in the apoptosis of FD BMSCs, cells were transfected with miR NC and miR-181a-5p mimics. Bcl-2 downregulation and Bax upregulation, with a decreased Bcl-2/Bax ratio, were observed in FD BMSCs transfected with miR-181a-5p mimics, which further confirmed apoptotic cell death (Fig. 2b). Immunofluorescence analysis also displayed the inhibited expression of Bcl-2 with miR-181a-5p overexpression (Fig. 2c). Moreover, we transfected BMSCs with a miR-181a-5p inhibitor to imitate FD BMSCs, and Bcl-2 expression and the Bcl-2/Bax ratio were increased (Fig. 2d, e). In addition, diminished BrdU incorporation was detected in FD BMSCs transfected with miR-181a-5p mimics, and the opposite results were observed in BMSCs treated with the miR-181a-5p inhibitor (Fig. 2f, g). These results suggest that downregulated miR-181a-5p suppresses apoptosis and promotes proliferation in FD BMSCs.

### Low expression of miR-181a-5p promotes osteogenic differentiation of BMSCs in FD

**Fig. 3:**
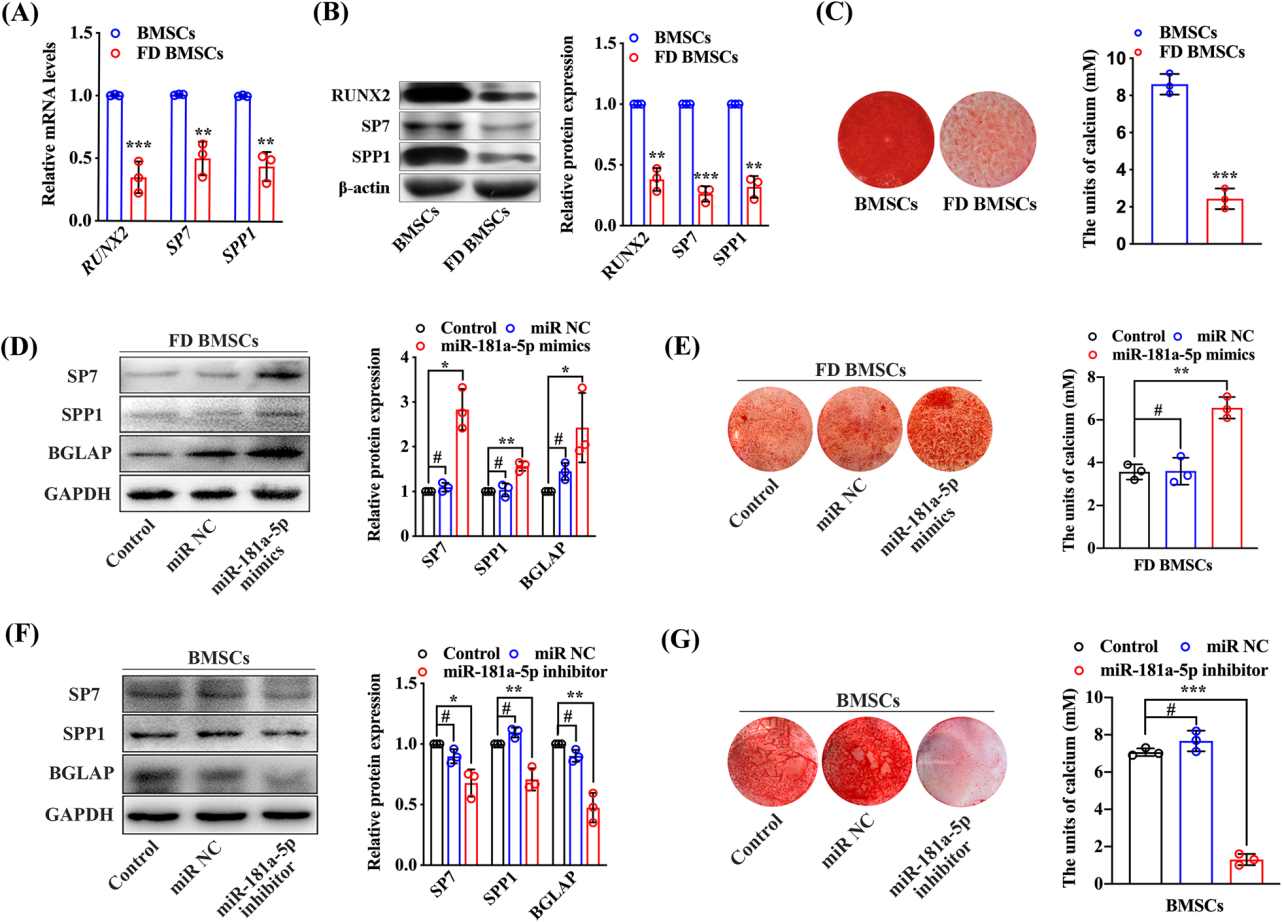
Low miR-181a-5p expression promotes osteogenic properties in FD BMSCs. **a** Quantitative real-time PCR analysis of osteogenesis-related marker mRNA expression in BMSCs and FD BMSCs after osteogenic induction for 7 days. **b** Western blot analysis of osteogenesis-related marker protein expression in BMSCs and FD BMSCs cultured with osteogenesis induction medium for 7 days. **c** Alizarin red staining and CPC assay showed calcium deposition in BMSCs and FD BMSCs after 14 days of osteogenic induction. Quantitative real-time PCR (**d**) and western blot (**e**) analysis of osteogenesis-related gene and protein expression in FD BMSCs transfected with miR-181a-5p mimics. Quantitative real-time PCR (**f**) and western blot (**g**) analysis of osteogenesis-related gene and protein expression in BMSCs transfected with the miR-181a-5p inhibitor. The data are presented as the mean ± S.E.M. values (n = 3). ^#^*P* > 0.05; *P < 0.05; **P < 0.01; ***P < 0.001

As our previous research reported, FD BMSCs exhibit weaker osteogenic capability than normal BMSCs (Xiao et al. 2019). In this study, the same result was shown (Fig. 3a–c). To confirm whether miR-181a-5p plays a role in osteogenesis, we transfected miR-181a-5p mimics into FD BMSCs and induced osteogenesis. FD BMSCs treated with miR-181a-5p mimics showed increased expression of osteogenic markers and increased calcium deposition formation (Fig. 3d, e), while opposite trends were noted after miR-181a-5p was inhibited in normal BMSCs (Fig. 3f, g). These results suggest that low miR-181a-5p expression impedes ossification of BMSCs in FD.

### Low miR-181a-5p expression in FD BMSCs boosts osteoclast differentiation

**Fig. 4:**
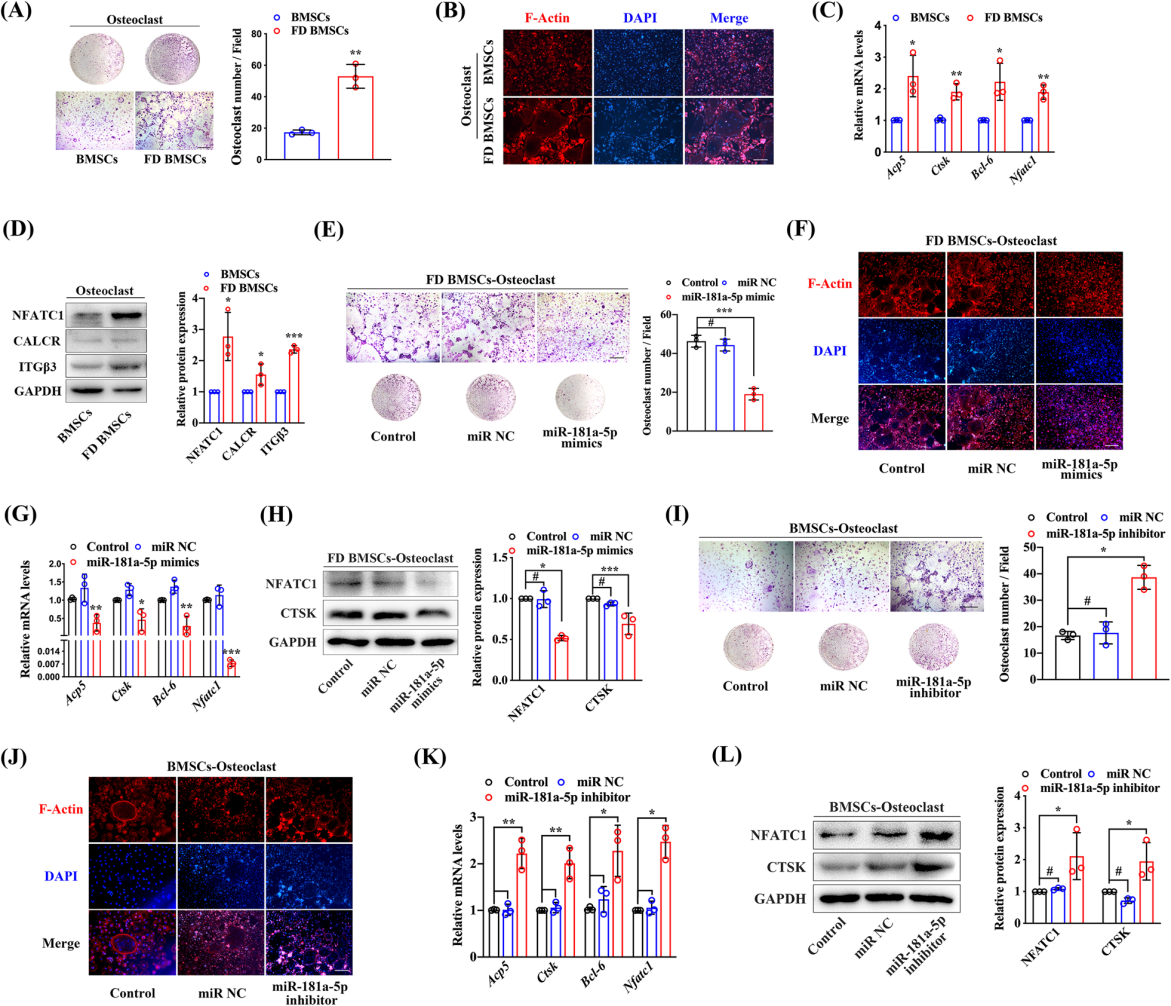
Low miR-181a-5p expression promotes FD BMSC-induced osteoclast differentiation and formation. **a** BMSC- and FD BMSC-induced osteoclast differentiation was analyzed by TRAP staining. **b** Representative image of the F-Actin ring structure in osteoclasts. Osteoclast differentiation-related gene expression was detected by quantitative real-time PCR (**c**) and western blot (**d**). **e** Effect of miR-181a-5p mimics on FD BMSC-induced osteoclast differentiation was analyzed by TRAP staining. **f** Representative image of the F-Actin ring structure in osteoclasts under miR-181a-5p mimics treatment. Osteoclast differentiation-related gene expression was detected by quantitative real-time PCR (**g**) and western blot (**h**) in osteoclasts induced by FD BMSCs transfected with miR-181a-5p mimics. **i** Effect of the miR-181a-5p inhibitor on BMSC-induced osteoclast differentiation was analyzed by TRAP staining. **j** Representative image of the F-Actin ring structure in osteoclasts under miR-181a-5p mimics treatment. Osteoclast differentiation-related gene expression was detected by quantitative real-time PCR (**k**) and western blot analysis (**l**) in osteoclasts induced by BMSCs transfected with miR-181a-5p inhibitor. Scale bar, 100 μm. The data are presented as the mean ± S.E.M. values (n = 3). ^#^*P* > 0.05; *P < 0.05; **P < 0.01; ***P < 0.001

Fibrous actin (F-Actin) rings are the characteristic cytoskeletal structures of osteoclasts and are essential for osteoclasts involving bone resorption (Jin et al. 2019). We compared the induced osteoclast differentiation between FD BMSCs and normal BMSCs, and the results exhibited more TRAP-positive multinucleated cells and more F-Actin ring formation in the FD BMSC supernatant group (Fig. 4a, b). Moreover, increased osteoclast differentiation markers were detected in the FD BMSC supernatant group (Fig. 4c, d). To identify the role of miR-181a-5p in osteoclast differentiation and formation, the supernatant of FD BMSCs transfected with miR-181a-5p mimics was collected and used to culture osteoclasts with RANKL and M-CSF. Decreased numbers of TRAP-positive multinucleated cells and fewer F-Actin rings were detected under miR-181a-5p mimic treatment (Fig. 4e, f), and the expression of osteoclast differentiation markers was also inhibited (Fig. 4g, h). Furthermore, miR-181a-5p inhibitor-treated BMSCs led to the opposite results (Fig. 4i–l). These results indicate that downregulated miR-181a-5p in FD BMSCs promotes osteoclast differentiation and formation, in turn boosting osteoclast activity.

### CREB1 interacts with miR-181a-5p by a feedback loop in FD BMSCs

**Fig. 5:**
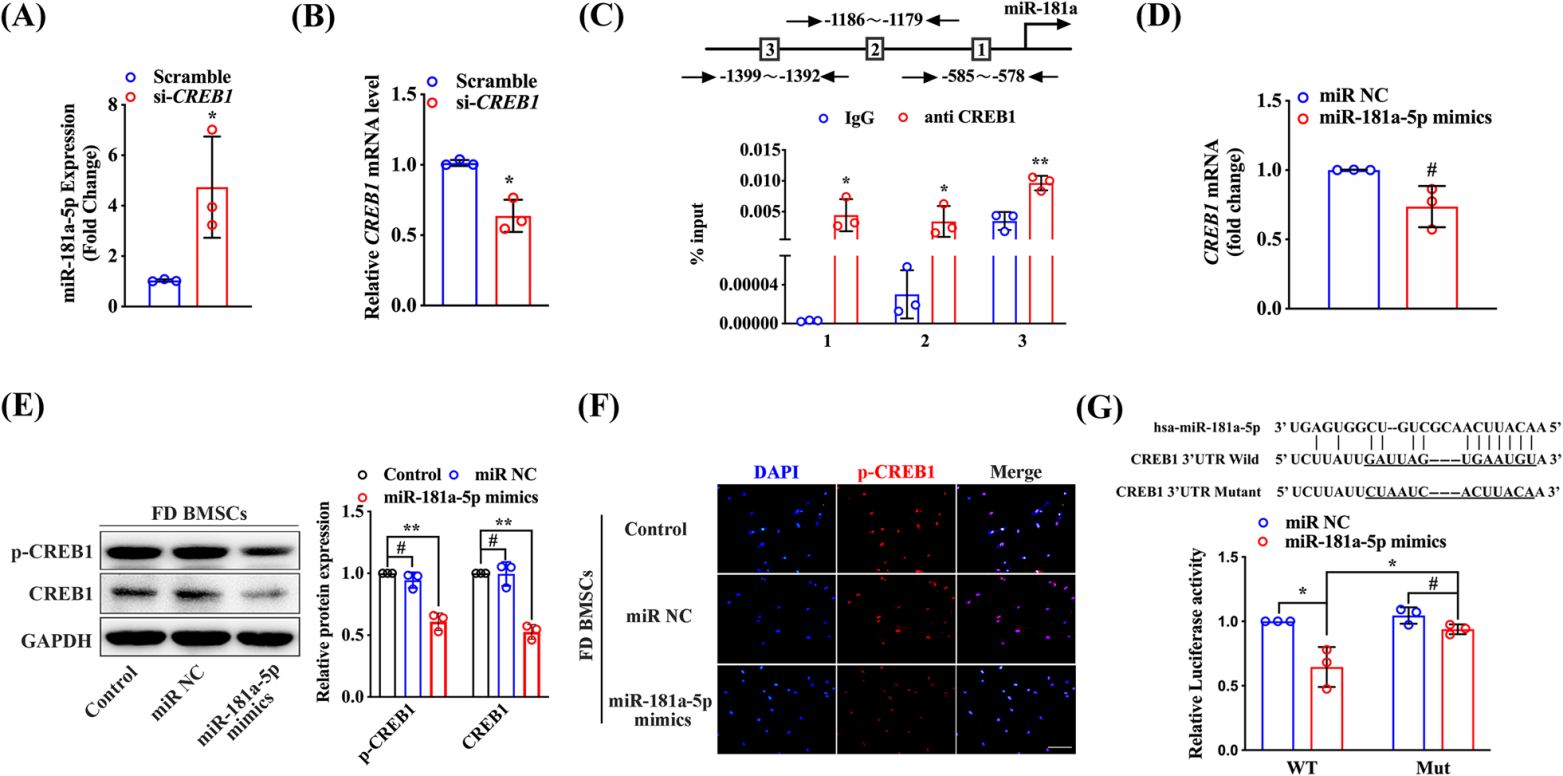
CREB1 interacts with miR-181a-5p through a feedback loop in FD BMSCs. After si-*CREB1* treatment for 48 h, CREB1 (**a**) and miR-181a-5p (**b**) in FD BMSCs were detected by quantitative real-time PCR. **c** Three primers were designed to cover the miR-181a promoter region and were used to identify CREB1 binding sites in the ChIP assay. All three putative CREB1 binding sites in the miR-181a promoter region were identified. **d** CREB1 mRNA expression was detected by real-time PCR in FD BMSCs transfected with miR-181a-5p mimics. **e** Protein expression of CREB1 and p-CREB1 was detected by western blot. **f** Immunofluorescence analysis of p-CREB1 expression in FD BMSCs transfected with miR-181a-5p mimics. **g** Effect of miR-181a-5p on a dual-luciferase reporter plasmid bearing wild-type (WT)/mutated (Mut) CREB1 binding sites was analyzed. Cells were cotransfected with either WT-CREB1 or Mut-CREB1 and miR-181a-5p mimics or miR NC. Firefly and Renilla luciferase activities were measured in cell lysates. Scale bar, 100 μm. The data are presented as the mean ± S.E.M. values (n = 3). *P < 0.05; **P < 0.01; ^#^*P* > 0.05

Based on the above observations, low expression of miR-181a-5p was detected in FD BMSCs, and exogenous cAMP suppressed miR-181a-5p expression in normal BMSCs. To confirm whether cAMP affected miR-181a-5p expression via CREB1, we used CREB1-targeting siRNA to transfect FD BMSCs (Fig. 5a). After CREB1-targeting RNAi, miR-181a-5p expression significantly increased (Fig. 5b). To further explore the regulatory roles of CREB1 on miR-181a-5p, we screened the miR-181a promoter region and found that CREB1 might bind to three putative binding sites (CREs) in the region from − 1399 to − 578 bp. ChIP assay with a specific anti-CREB1 construct and three primers covering the miR-181a promoter region were performed. Significant enrichment of CREB1 was observed at all three putative binding sites in the miR-181a promoter region (Fig. 5c). Furthermore, we transfected miR-181a-5p mimics into FD BMSCs and found that the CREB1 mRNA level had no change, whereas the p-CREB1 protein level was decreased after CREB1 reduction (Fig. 5d, e). Immunofluorescence analysis also displayed the downregulation of p-CREB1 with miR-181a-5p overexpression (Fig. 5f). To further verify the regulatory function of miR-181a-5p on CREB1, we constructed CREB1 luciferase reporter plasmids encoding the predicted 3’UTR of CREB1 mRNA in wild-type (WT) or site-mutated (Mut) forms. The reporter was cotransfected with miR NC or miR-181a-5p mimics into 293T cells. The results from the luciferase reporter assay indicated that the luciferase activity significantly decreased in cells with the reporter comparable to that of miR NC (Fig. 5g). Collectively, these findings indicate that CREB1 inhibits miR-181a transcription by directly binding to its promoter region, while miR-181a-5p binds to CREB1 through a posttranscriptional repression mechanism and further verify that CREB1 interacts with miR-181a-5p by a feedback loop in FD BMSCs.

## Discussion

Fibrous dysplasia (FD) is a slow-progressing BMSC-related disease with the main pathological feature being the replacement of normal bone tissue by immature fibrous trabeculae (Michienzi et al. 2007; Robey et al. 2007). FD in cranial maxillofacial bones usually has no obvious symptoms in the incipient stage. With progression of the disease, local swelling deformity, occlusal disorder, pathological fracture, local pain and other symptoms often present clinically. Mutations in GNAS are the critical cause of FD. We recently reported that compared to normal BMSCs, FD BMSCs demonstrate weaker apoptotic and osteogenic differentiation abilities and a stronger proliferation ability (Florenzano et al. 2019). In addition, FD BMSCs exhibit high osteoclastic activity in mouse models (Zhao et al. 2018; Castro et al. 2019). However, the detailed mechanism underlying these clinical features remains unclear.

miRNAs are endogenous noncoding small RNA molecules that are cut from miRNA precursors (pre-miRNAs). Mature miRNAs can form complexes with RISC and then bind to target mRNA sites through base pairing to regulate target gene expression (Treiber et al. 2019). In mammals, miRNAs are not completely complementary to the mRNA 3'UTR of target genes to inhibit their protein expression at the translation level (Gebert and MacRae 2019). Existing studies have shown that miRNAs are involved in a series of biological processes, such as cell proliferation and differentiation. A recent report identified a number of circulating miRNAs that are potential negative regulators of gene expression in bone cell progenitors and were associated with FD (Legrand et al. 2020). In this study, we performed miRNA sequencing analysis of normal BMSCs and FD BMSCs, and the results showed that miR-181a-5p, miR-145-3p, miR-98-3p and miR-92b-5p were significantly differentially expressed miRNAs. Down-regulated expression of miR-181a-5p and miR-145-3p and up-regulated expression of miR-92b-5p with cAMP or IBMX treatment suggested the possible relationship between either miR-181a-5p, miR-145-3p or miR-92b-5p and FD, while up-regulated of miR-98-3p might cause by other regulatory mechanism. Since miR-181a-5p was found that associating with apoptosis, osteogenic differentiation and osteoclast differentiation, we selected miR-181a-5p, which was the most significant downregulated miRNA in FD BMSCs (fold > 10, *P* < 0.05), and further explored its function in FD (Bhushan et al. 2013; Ouyang et al. 2012; Wang et al. 2014).

miR-181a belongs to a very conserved miRNA family. Previous studies have shown that miR-181 family members can complement multiple target miRNAs and participate in the regulation of pathophysiological processes of various diseases (Braicu et al. 2019; Seoudi et al. 2012) (See Footnote 1). Recent studies have found that miR-181a can induce apoptosis in senescent cells by inhibiting the protein expression of the target gene B-cell leukemia 2 (Bcl-2) (Rippo et al. 2014). In addition to this activity in senescent cells, miR-181a also promotes apoptosis in other cells by regulating Bcl-2 (Zhu et al. 2012; Chen et al. 2010). A large number of studies have shown that mitochondrial pathways play a crucial role in apoptosis, among which Bcl-2 and BCL2-associated X (Bax), a member of the Bcl-2 family, are important regulatory genes that play an opposite role in the process of apoptosis (Adams and Cory 2007; Garner et al. 2019; Cheng et al. 1997). A decrease in the Bcl-2/Bax ratio was associated with apoptotic cell death with activation of caspase-3 and cleavage of PARP (Karna et al. 2009). An increased Bcl2/Bax ratio contributes to enhanced survival and proliferation (Zhang et al. 2012b). In the current study, Bcl-2 overexpression was detected in FD BMSCs, while Bax was downregulated. Studies have confirmed that the formation of the Bcl-2/Bax heterodimer can inhibit the occurrence of apoptosis by inhibiting the activation of downstream caspase-3 (Cheng et al. 1997). Considering the lower expression levels of activated caspase-3 and PARP proteins in FD BMSCs reported in our previous study (Xiao et al. 2019), we infer that high expression of Bcl-2 and a high Bcl-2/Bax ratio may be the key reason for the inhibition of FD BMSC apoptosis and further indicate that downregulated miR-181a-5p contributes to Bcl-2 upregulation in FD BMSCs.

The occurrence of FD is related to the broken balance of osteogenic and osteoclastic activities. Our previous results showed that the osteogenic differentiation and mineralization capacity of FD BMSCs were weaker than those of normal BMSCs (Xiao et al. 2019). Furthermore, we found that the number of osteoclasts induced by the FD BMSC supernatant increased and that the expression of osteoclast-related genes was enhanced, suggesting that pathological fracture in FD patients might be related to enhanced activity of osteoclasts. In terms of bone metabolism, miR-181a can regulate BMP-induced osteogenic differentiation of MC3T3 cells by inhibiting the TGF-beta pathway (Bhushan et al. 2013), and miR-181a has an inhibitory effect on osteoclast survival (Wang et al. 2014). Interestingly, miR-181a promoted osteoclast apoptosis by regulating FasL protein expression (Shao et al. 2015). Our study showed that FD BMSCs treated with exogenous miR-181a-5p exhibited enhanced osteogenic differentiation and mineralization abilities and weaker osteoclast activity. These data imply that a sufficient miR-181a level is an important factor in maintaining bone homeostasis.

The regulatory effect of cAMP on cells is realized by activating the downstream PKA-CREB pathway. Phosphorylated CREB forms a homodimer that binds to a class of cAMP response element (CRE) in the gene transcriptional regulatory region to regulate gene transcription. Some miRNAs may have independent transcriptional regulatory units and in the same cluster are generally co-transcribed (Ha and Kim 2014; Stavast and Erkeland 2019), and transcription factors could positively or negatively regulate miRNA expression (Ha and Kim 2014). Recent studies have demonstrated that CREB1 can act with miRNAs in regulatory networks by feedback loops (Wang et al. 2016). In this study, we uncovered that miR-181a-5p was regulated by CREB1. To gain further insight into the mechanism, we analyzed the promoter region of miR-181a and identified three putative binding sites of CREB1. Our data first verified that miR-181a-5p is the downstream effector of CREB1. However, the detailed mechanism remains largely unexplored and needs to be further investigated.

The regulation of target genes by miRNAs mainly occurs at the posttranscriptional level. In animals, most miRNAs regulate the expression of target genes mainly by complementing the target gene mRNA 3'UTR to suppress gene translation (Gebert and MacRae 2019). MiR-181a regulates CREB1 expression by targeting its mRNA 3'UTR in neurons (Liu et al. 2013). In our study, we found that miR-181a-5p inhibited the protein but not the mRNA expression of CREB1 in FD BMSCs and further ruled out that miR-181a-5p regulated CREB1 expression by binding to its 3'UTR. These results suggest that miR-181a-5p has a negative regulatory effect on CREB1 expression by translation suppression. The presence of the CREB1-miR-181a-5p loop, in which CREB1 interacts with miR-181a-5p by negative feedback regulation, is critically involved in the abnormal properties of FD BMSCs.

**Fig. 6:**
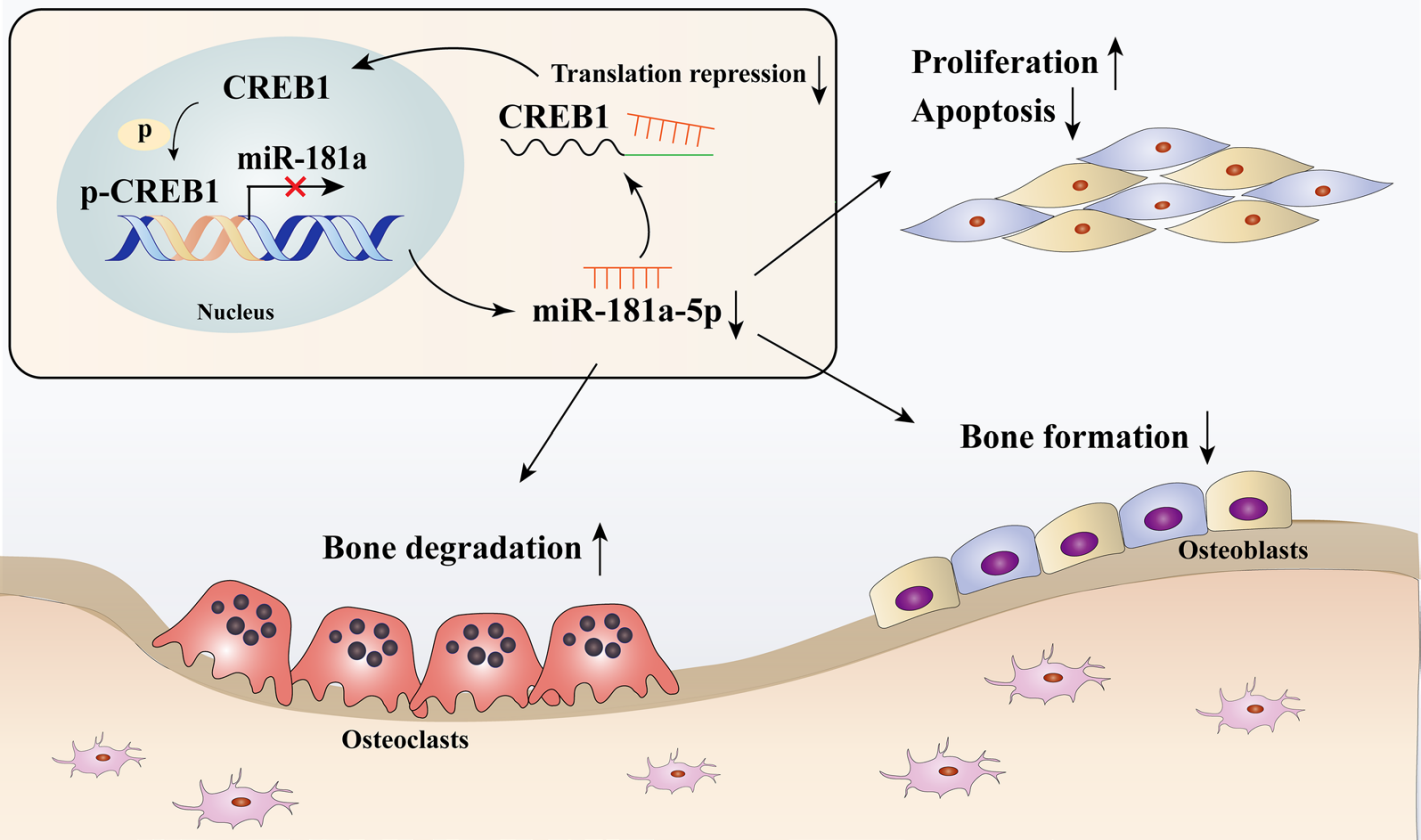
The CREB1-miR-181a-5p loop in BMSCs from FD lesions. Overproduction of cAMP promotes CREB1 phosphorylation, and p-CREB1 binds to the CRE in the miR-181a promoter to suppress its transcription. Downregulation of miR-181a-5p leads to enhanced proliferation ability, decreased apoptotic capacity, and impaired osteogenic differentiation of FD BMSCs and increases the osteoclast differentiation potential, resulting in clinicopathological features of FD. Moreover, downregulation of miR-181a-5p promotes CREB1 expression by decreasing the inhibition of its translation and further exacerbates pathological manifestations of FD

In summary, our studies, taken together with these results, demonstrate for the first time that the CREB1-miR-181a-5p loop is associated with the disordered properties of FD BMSCs (Fig. 6). Furthermore, intervention with miR-181a-5p reversed the FD phenotype to some extent. Importantly, this study provides useful insights into the molecular pathogenesis of FD and the evaluation of potential therapeutic strategies for FD in vitro.

## Conclusions

The current study demonstrates that the CREB1-miR-181a-5p loop participates in the pathological phenotype of FD BMSCs. In this context, our results suggest that targeted modulation of miR-181a-5p in FD BMSCs might be a potential therapeutic strategy for FD in vitro. Continued investigation of this loop may be promising for providing a possible therapeutic strategy for FD patients.

## Supplementary Information

**Additional file 1.** **Table S1.** The primary antibodies used in western blot.

**Additional file 2.** **Table S2.** Sequence information on specific primers used in this study.

**Additional file 3.** **Table S3.** Sequence of ChIP primers.

## Data Availability

The datasets used and/or analyzed during the current study are available from the corresponding author on reasonable request.

## Abbreviations

BMSCs: Bone marrow stromal cells
FD: Fibrous dysplasia
miRNA: MicroRNA
GNAS: Guanine nucleotide-binding protein alpha-stimulating activity polypeptide
cAMP: Cyclic adenosine monophosphate
CREB: CAMP-response element-binding protein
PKA: CAMP-dependent protein kinase
CRE: CAMP-response element
mRNA: Messenger RNA
DMEM: Dulbecco’s Modified Eagle Medium
FBS: Fetal bovine serum
KEGG: Kyoto Encyclopedia of Genes and Genomes
CPC: Cetylpyridinium chloride
BMMCs: Bone marrow mononuclear cells
BrdU: Bromodeoxyuridine
IBMX: 3-Isobutyl-1-methylxanthine
TRAP: Tartrate-resistant acid phosphate
ChIP: Chromatin immunoprecipitation
