# Supplementary material for: A CREB1-miR-181a-5p loop regulates the pathophysiologic features of bone marrow stromal cells in fibrous dysplasia of bone
Source: Mol Med. 2021 Jul 22;27:81. doi: 10.1186/s10020-021-00341-z (PMC8296714; doi:10.1186/s10020-021-00341-z)
Supplement: Supplementary file 1 — Additional file 1. Table S1. The primary antibodies used in western blot. [file 10020_2021_341_MOESM1_ESM.docx]

Table S1:The primary antibodies used in western blot.

| Antibody | Dilution | Source | Code number |
| --- | --- | --- | --- |
| RUNX2 | 1:1000 | Cell Signaling Technology | #12556 |
| SP7 | 1:1000 | Abcam | Ab22552 |
| SPP1 | 1:1000 | Proteintech | 25715-1-AP |
| BGLAP | 1:800 | Bioworld | BS60987 |
| ITGβ3 | 1:1000 | Affinity | AF6085 |
| NFATc1 | 1:1000 | Cell Signaling Technology | #8032 |
| CALCR | 1:1000 | Proteintech | 20868-1-AP |
| CREB | 1:1000 | Cell Signaling Technology | #9197 |
| p-CREB | 1:1000 | Cell Signaling Technology | #9198 |
| Bcl-2 | 1:1000 | Proteintech | 12789-1-AP |
| Bax | 1:1000 | Proteintech | 50599-2-lg |
| GAPDH | 1:1000 | Proteintech | 60004-1-lg |
