# Supplementary material for: A CREB1-miR-181a-5p loop regulates the pathophysiologic features of bone marrow stromal cells in fibrous dysplasia of bone
Source: Mol Med. 2021 Jul 22;27:81. doi: 10.1186/s10020-021-00341-z (PMC8296714; doi:10.1186/s10020-021-00341-z)
Supplement: Supplementary file 2 — Additional file 2. Table S2. Sequence information on specific primers used in this study. [file 10020_2021_341_MOESM2_ESM.docx]

Table S2:Sequence information on specific primers used in this study.

| Gene | Primer Sequence (5’ - 3’) |
| --- | --- |
| *RUNX2* | F:5’-TCACCTCAGGCATGTCCCTCGGTAT-3’  R:5’-TGGCTTCCATCAGCGTCAACACC-3’ |
| *SP7* | F: 5’-AACCCCCAGCTGCCCACCTACC-3’  R: 5’-GACGCTCCAGCTCATCCGAACG-3’ |
| *SPP1* | F:5’-ATGGAAAGCGAGGAGTTGAATG-3’  R:5’-TGCTTGTGGCTGTGGGTTT-3’ |
| *Nfatc1* | F:5’-CAACGCCCTGACCACCGATAG-3’  F:5’-GGCTGCCTTCCGTCTCATAGT-3’ |
| *Acp5* | F:5’-CAGCCCTTATTACCGTTTGC-3’  R:5’-GAATTGCCACACAGCATCAC-3’ |
| *Ctsk* | F:5’-GGGAGAAAAACCTGAAGC-3’ |
|  | F:5’-ATTCTGGGGACTCAGAGC-3’ |
| *Bcl-6* | F:5’-TCCAGTCCCCACTCACTCAC-3’  F:5’-TTGCTCAAAACCAAATGAGCACT-3’ |
| *Gapdh* | F:5’-ACAACTTTGGTATCGTGGAAGG-3’  R:5’-GCCATCACGCCACAGTTTC-3’ |
| *U6* | F: 5’-CTCGCTTCGGCAGCACATATACT-3’ |
|  | R: 5’-ACGCTTCACGAATTTGCGTGTC-3’ |
| miR-181a-5p | 5’-AAGCGGAACATTCAACGCTGTCG-3’ |
| Universal primers | 5’-ATCCAGTGCAGGGTCCGAGG-3’ |
