# Supplementary material for: A CREB1-miR-181a-5p loop regulates the pathophysiologic features of bone marrow stromal cells in fibrous dysplasia of bone
Source: Mol Med. 2021 Jul 22;27:81. doi: 10.1186/s10020-021-00341-z (PMC8296714; doi:10.1186/s10020-021-00341-z)
Supplement: Supplementary file 3 — Additional file 3. Table S3. Sequence of ChIP primers. [file 10020_2021_341_MOESM3_ESM.docx]

Table S3:Sequence of ChIP primers.

| Numbers | Primer Sequence (5’ - 3’) | Sites |
| --- | --- | --- |
| miR-181a promoter-1 | F: 5’-GGCAACACCCCATGTCATTCC-3’  R: 5’- AGCGATGTCAAGCCTTATACTATTTGG-3’ | -646 ~ -456 |
| miR-181a promoter-2 | F: 5’-TGGTATCGTTTCAGCCAGAACAA-3’  R: 5’-TCTCCTTTGCTGAGATCCTGCA-3’ | -1352 ~ -1155 |
| miR-181a promoter-3 | F: 5’-TGCAGGATCTCAGCAAAGGAGA-3’  R: 5’-AGACCCATCACTGCAGAGCC-3’ | -1482 ~ -1331 |
